# Medial patellofemoral ligament reconstruction normalizes patellar kinematics but fails to predict cartilage contact area: A prospective 3D MRI study

**DOI:** 10.1002/jeo2.70119

**Published:** 2024-12-30

**Authors:** Markus Siegel, Philipp Maier, Elham Taghizadeh, Hans Meine, Thomas Lange, Andreas Fuchs, Tayfun Yilmaz, Hagen Schmal, Kaywan Izadpanah

**Affiliations:** ^1^ Department of Orthopedic Surgery and Traumatology, Freiburg University Hospital Albert Ludwigs University Freiburg Freiburg Germany; ^2^ Fraunhofer Institute for Digital Medicine MEVIS Bremen Germany; ^3^ Division of Medical Physics, Department of Diagnostic and Interventional Radiology Medical Center, Faculty of Medicine University of Freiburg Freiburg Germany; ^4^ Department of Orthopedic Surgery University Hospital Odense Odense C Denmark

**Keywords:** dynamic, knee, MPFL, MRI, patellofemoral

## Abstract

**Introduction:**

The medial patellofemoral ligament (MPFL) is the main patellar stabilizer in low knee flexion degrees (0–30°). Isolated MPFL reconstruction (MPFLr) is therefore considered the gold standard of surgical procedures for low flexion patellofemoral instabilities (PFIs). Despite excellent clinical results, little is known about the effect of MPFLr on kinematic parameters (KPs) of the patellofemoral joint in vivo. This study investigates the effect of MPFLr on KP of patellofemoral articulation, using a three‐dimensional (3D) in vivo magnetic resonance imaging (MRI) analysis at different flexion and loading positions, and analyzes the correlation of these parameters with the patellofemoral cartilage contact area (CCA).

**Methods:**

In this prospective, matched‐pair cohort study of 30 individuals, 15 patients with low flexion PFI and 15 knee‐healthy individuals were included. Patients were analyzed pre and post‐operatively after MPFLr. MRI images were obtained at 0°, 15° and 30° with and without muscle activation, using a custom‐designed pneumatic loading device. Patellar shift, tilt and rotation were determined in 3D bone and cartilage models of each individual, guaranteeing the highest reliability. Subsequently, the KPs were correlated with patellofemoral CCA.

**Results:**

Patients with low flexion PFI had a leg geometry of 0.5 ± 2.6° valgus and a TTTG of 11.4 ± 4.4 mm. Eleven patients had moderate (Type A/B) and 2 had severe (Type C/D) trochlear dysplasia. Without muscle activation, patients showed significantly increased patellar shift (0–30°; *p*_0°_ = 0.011, *p*_15°_ = 0.004 and *p*_30°_ = 0.015) and tilt (15°; *p*_15°_ = 0.041). Muscle activation did not compensate for maltracking in these patients, but even increased tilt and shift further in extension (*p*_0°_ = 0.002 and *p*_0°_ = 0.001). MPFLr statistically reduced patellofemoral tilt from 0° to 30° flexion during passive flexion and tended to approach the values of knee‐healthy individuals (*p*_ext_ = 0.008, *p*_15°_ = 0.006 and *p*_30°_ = 0.003). Post‐operatively, muscle activation led to comparable tilt and shift as in healthy individuals. Tilt, shift and rotation did not correlate with CCA neither in healthy individuals nor in pre‐ or post‐operative patients.

**Conclusion:**

Isolated MPFLr can normalize patellar shift and tilt in patients with low flexion instability. Considering the influence of muscle activation, passive stabilization through MPFLr seems to be the basic precondition for physiologically active patella stabilization. The investigated KPs as easy‐to‐measure parameters in clinical practice cannot be used to assume normalized CCA for low flexion degrees. Therefore, methodologically demanding methods are still required to calculate the patellofemoral CCA.

**Level of Evidence:**

Level II.

**Abbreviations**
3D: three‐dimensional
BMI: body mass index
CCA: cartilage contact area
Fig: figure
KP: kinematic parameter
MPFL: medial patellofemoral ligament
MPFLr: medial patellofemoral ligament reconstruction
MRI: magnetic resonance imaging
PFI: patellofemoral instability
PFJ: patellofemoral joint
TCA: transcondylar axis
TEA: transepicondylar axis
TTTG: tibial tuberosity to trochlear groove

## INTRODUCTION

Patellofemoral instability (PFI) is a common clinical entity, especially in adolescents, and is considered one of the main risk factors for the development of knee pain [17]. In up to 95% of patients, patellar luxation leads to cartilage damage and thus significantly increases the risk of osteoarthritis [18, 21]. Among young, active subjects, both the prevalence (up to 33/100,000) and the risk of recurrent dislocation under conservative therapy (up to 70%) are highest and are among the most debilitating diseases of the knee joint in this population [8, 10, 14]. Despite the high prevalence, the underlying biomechanical causes are well‐researched, but the biomechanical characteristics are not completely understood [20]. The stabilization of the patellofemoral joint (PFJ) is complex and is determined by the bony geometry of the PFJ (congruency), the passive soft tissue structures, and the dynamic/active muscle function [4]. Most importantly, the medial patellofemoral ligament (MPFL) contributes significantly to patellofemoral stabilization, with approximately 50%–60% of the total restraining forces during low (0–30°) knee flexion [5]. Moreover, the influence of the quadriceps muscle increases steadily within the first 30° of knee flexion and is considered an active stabilizer of the patella [3, 12]. In surgical therapy, isolated MPFL reconstruction (MPFLr) is widely used for the treatment of PFI in the absence of concomitant pathologies. This is due to its high success rate, with reluxation rates ranging from 0% to 4.5% [6, 7, 11]. The determination of the kinematic patellofemoral parameters before and after MPFLr, based on in vivo data, is important for drawing conclusions about the patellofemoral articulation and enabling the best patient‐specific therapy. Recently, it has been proven that the patellofemoral kinematics and the patellofemoral contact of patients with PFI depend on different factors. Besides the trochlear geometry, a reduced influence of the quadriceps musculature on the patellofemoral contact mechanism is evident in patients with PFI [9, 25]. Despite the fact that MPFLr has been shown to restore patellofemoral cartilage contact area (CCA) and ensure patellofemoral stability, a relevant incidence of patellofemoral osteoarthritis has been reported in the further course [24, 27, 28]. The determination of the exact individual patellofemoral kinematics in vivo with and without muscle activation has not yet been fully clarified and therefore requires further attention.

The present study investigates patellofemoral kinematics (patellar shift, tilt and rotation) in patients with low‐flexion PFI (patellar instability in the range from 0° to 30° of flexion) before and after patellofemoral stabilizing surgery by MPFLr. It uses high‐resolution three‐dimensional (3D) magnetic resonance imaging (MRI) data sets acquired in different knee flexion positions (0–30°) with and without muscle activation. It also analyzes the correlation of the kinematic parameters (KPs) to the CCA across the different flexion angles and loading situations.

We hypothesize that MPFLr will alter KPs both with and without muscle activation.

## MATERIALS AND METHODS

### Subjects and diagnostic methods

In this prospective, matched cohort study, a cohort of 30 individuals, consisting of 15 patients with low‐flexion PFI and 15 matched volunteers with healthy knee joints, was evaluated using MRI scans with static loading. Patients were analyzed pre‐ and post‐operatively after MPFLr using gracilis tendon autografts. Inclusion criteria for patients were clinically manifest low flexion PFI (0–30° knee flexion), age between 18 and 65 years, and no history of surgical intervention at the patellofemoral joint. Patients were recruited from the waiting list for patellofemoral stabilizing surgery via MPFLr. PFI was diagnosed based on clinical symptoms, including patellofemoral pain, subjective and objective clinical instability using the glide test and patellar apprehension test, and a history of recurrent patellofemoral dislocation events. For the selection of therapy, we based our decision on the clinical presentation of low‐flexion patellofemoral instability (PFI). For patients presenting with flexion‐related PFI (instability at higher degrees of flexion), additional treatment options such as trochleoplasty may be appropriate, particularly in cases of trochlear dysplasia with relevant geometric and contextual considerations. However, these patients were excluded from the study cohort.

Each patient also received standard projection radiography and an MRI scan of the knee joint to confirm the diagnosis. The MPFLr was performed using autologous gracilis tendons. The femoral position was defined according to Schöttle et al. and fixation to the patella was performed with two suture anchors [22].

Patients were excluded if they had undergone knee surgery in the past, had existing metal implants, retropatellar arthrosis or chondromalacia of the patella, suffered from claustrophobia, did not meet the age requirements, or were pregnant.

### MRI setup and protocol

MRI was performed preoperatively and 11 ± 5 weeks post‐operatively using a Magnetom Trio 3 T system (Siemens Healthineers) and an eight‐channel multipurpose coil (NORAS MRI products), which was attached to the subject's thigh using a hook‐and‐loop fastener. The knee flexion angle was adjusted with a knee support. A dedicated MR‐compatible pneumatic loading device enabled axial knee loading in the range of 0–500 N by applying pressure against the sole of the foot and could be controlled from the console room [13]. Such loading with knee flexion was supposed to give rise to quadriceps muscle activation.

**Figure 1:**
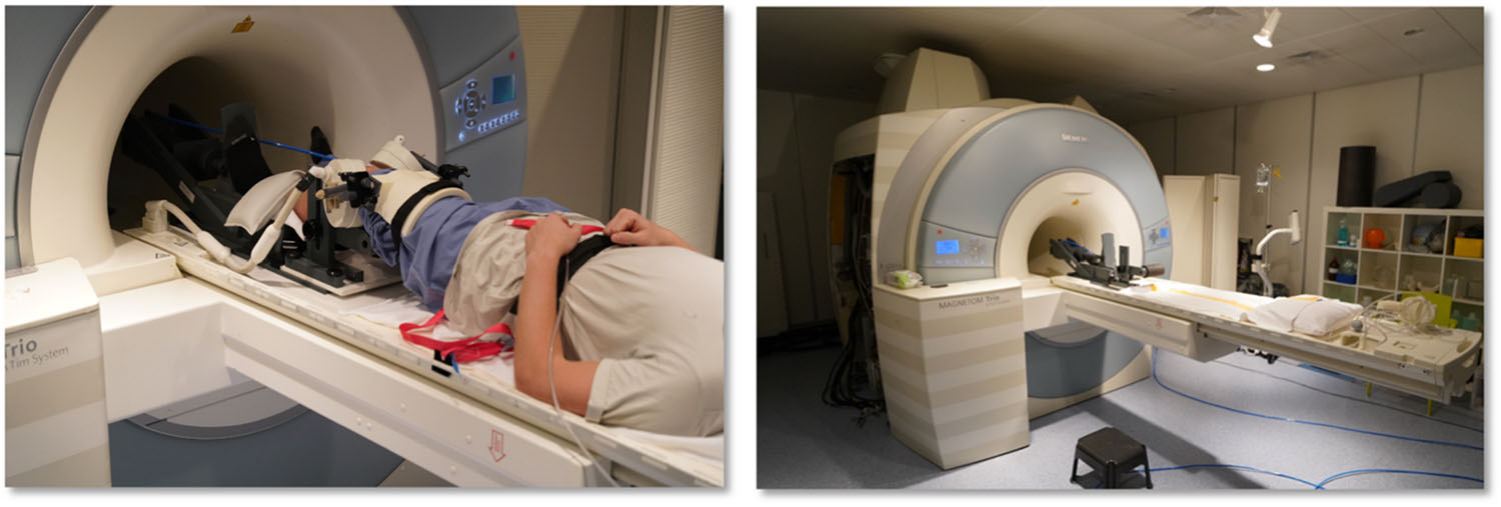
Experimental setup for the MRI examinations (left) with a pneumatic loading device (right). MRI, magnetic resonance imaging.

For safety reasons, the subjects were given an emergency switch, with which they could release the load instantaneously. The subjects were fixed on the scanner bed with a weight‐lifting belt. For the examination, the leg was placed on the carriage of the pneumatic knee loading device (Figure 1). Measurements were performed at 0°, 15° and 30° knee flexion angles. Initially, the measurements were performed without mechanical load, then subsequently with a load of 50 N. A 3D turbo spin echo protocol with parallel imaging (Generalized Autocalibrating Partially Parallel Acquisition) acceleration by a factor of 2 and an isotropic resolution of 0.5 mm was used for the MRI scans. Other scan parameters were TR = 1.8 s, TE = 59 ms, receiver bandwidth = 504 hz/Px and scan duration = 6.20 min. The 3D measurement volume was positioned to cover the entire PFJ. Load‐induced motion artefacts were mitigated via prospective motion correction [21] using a moiré phase tracking system (Metria Innovation) [22]. This system consisted of a tracking camera mounted on top of the scanner bore and a single tracking marker attached to the subject's knee cap. The tracking system captured both translational and rotational movements of the marker at a rate of 80 frames per second. Using the optically acquired motion data, a real‐time update of the MRI measurement volume was performed prior to each excitation pulse of the MRI sequence. Motion correction between scans (position locking) was used to ensure that unloaded and loaded MRI scans were performed with the originally planned field of view.

**Figure 2:**
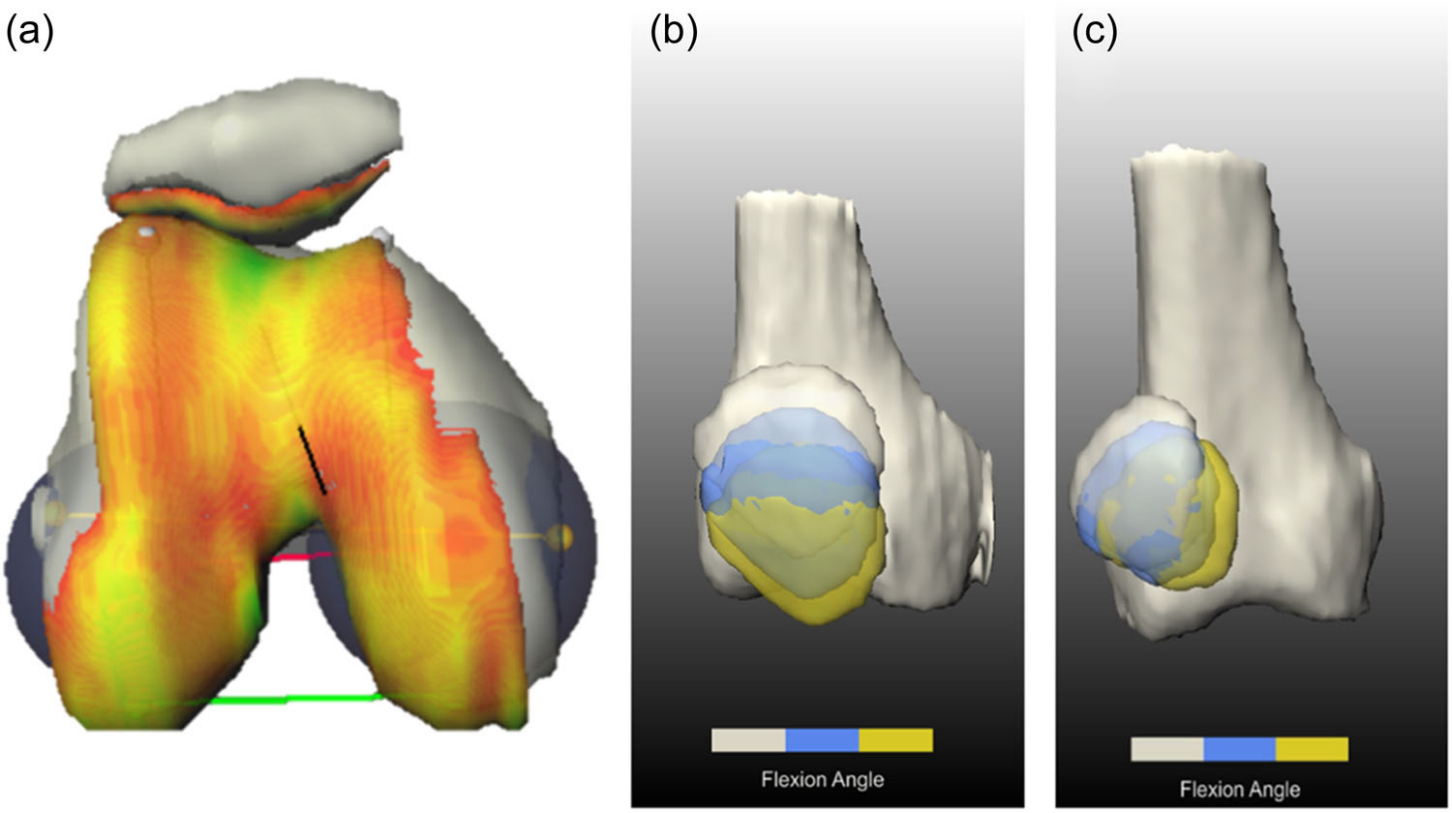
(a) 3D bone and cartilage mesh, (b) 3D bone mesh of a healthy subject with interposition of the patellar over the different flexion positions (grey 0° of flexion, blue 15° of flexion and yellow 30° of flexion), (c) 3D bone mesh of a patient with low flexion PFI (grey 0° of flexion, blue 15° of flexion and yellow 30° of flexion). 3D, three‐dimensional.

Further data processing and quantification were performed using the SATORI image processing platform, developed by Fraunhofer MEVIS and based on MeVisLab. The bones and cartilage of the entire cohort were first segmented entirely manually. 3D meshes of individual structures were generated from the segmented structures, which were then transferred to all other flexion situations (0°, 15° and 30° without and with 50 N loading, respectively) using image registration. The weight load of 50 N was chosen to activate the quadriceps muscles and to overcome individual muscular differences with the aim of generating a robust method that avoids severe movement artefacts. Image registration was performed on a limited area of each bone individually, optimizing the normalized gradient field distance of the MRI images. Based on the assumption that the bones do not deform significantly, this creates an individual surface mesh for each bone. The individually generated 3D bone profiles thus articulate with each other and can be used for the further analysis of patellofemoral kinematics (Figure 2).

In this way, patellofemoral KPs could be determined for each study participant in all three flexion positions with and without muscle activation. The CCA was defined as the patellofemoral cartilage area in which the Euclidean distance between the two opposing cartilage surfaces was less than 1 mm. We carried out the post‐operative measurement when the knee joint was clinically free of irritation and full weight‐bearing was possible in order to guarantee a measurement with weight‐bearing.

### Kinematic parameters

#### Patella rotation

Patella rotation was defined as the movement of the patella around the anterior–posterior axis of the femur [31]. Lateral rotation (positive rotation) describes the external rotation of the distal patellar pole relative to the proximal patellar pole; consequently, medial rotation (internal rotation) is indicated by negative values.

#### Patella shift

Mediolateral translation (lateralization) of the patella is defined as a transverse movement of the patella centre along a mediolateral axis of the femur or the patella itself [1, 29]. We defined patellar shift as the movement of the patella along the transcondylar axis (TCA). Medial translation is considered a negative shift, and lateral translation is considered a positive shift [31]. TCA was determined individually for each study participant, based on the 3D mesh.

#### Patella tilt

The patellar tilt is defined as the rotation of the patella around the longitudinal axis of the femur or patella. If the patella tilts laterally, this is called a positive tilt, in which the lateral edge of the patella rotates in the direction of the medial condyle of the femur [31].

### Statistics

A matched‐pair analysis was performed to compare the groups while controlling for potential confounding factors. Pairs were matched based on sex and knee size, measured by the transepicondylar axis (TEA) distance, to ensure comparability between the groups. This approach aimed to minimize the influence of confounders and improve the validity of the comparison.

Descriptive statistics are reported in terms of means and standard deviations. Differences within the subgroups were analyzed using the Wilcoxon signed‐rank test. The Wilcoxon rank‐sum test was used to compare the different cohorts. Correlations were calculated using the Spearman correlation test and reported using *r* coefficients. Correlation was characterized as poor *r* < 0.3, weak *r* = 0.31–0.5, moderate *r* = 0.51–0.7 and strong *r* > 0.71. A *p* < 0.05 was considered statistically significant.

Statistical analyses were performed using IBM SPSS Statistics version 28.0.0.0 (IBM Corp.). The results of all the statistical tests were interpreted in an exploratory sense. Consequently, the *p* values and 95% confidence intervals were not corrected for multiple comparisons, and the measurements may not be reproducible.

## RESULTS

In total, the data sets of 30 subjects, 15 patients with low‐flexion PFI and the data sets for 15 subjects with healthy knees, could be included.

### Participants and selection process

Twenty patients with clinically manifest PFI were identified preoperatively and could be recruited for this study to participate in the MRI examinations. Due to a problem with the MRI scanner and a technical malfunction of the collected data set, two study participants could not be included for further analysis. One patient was eliminated due to previously undiagnosed claustrophobia. Seventeen patient scans were suitable for preoperative analysis. However, after their preoperative scans, one patient decided against surgery, and another measurement could not be considered for further analysis due to severe artefacts, which made the transfer of the registration impossible. Another patient showed post‐operative joint effusion, which is why the values of the CCA in 0° and 15° could not be included in the calculation.

This meant that a total of 15 recordings were included.

**Figure 3:**
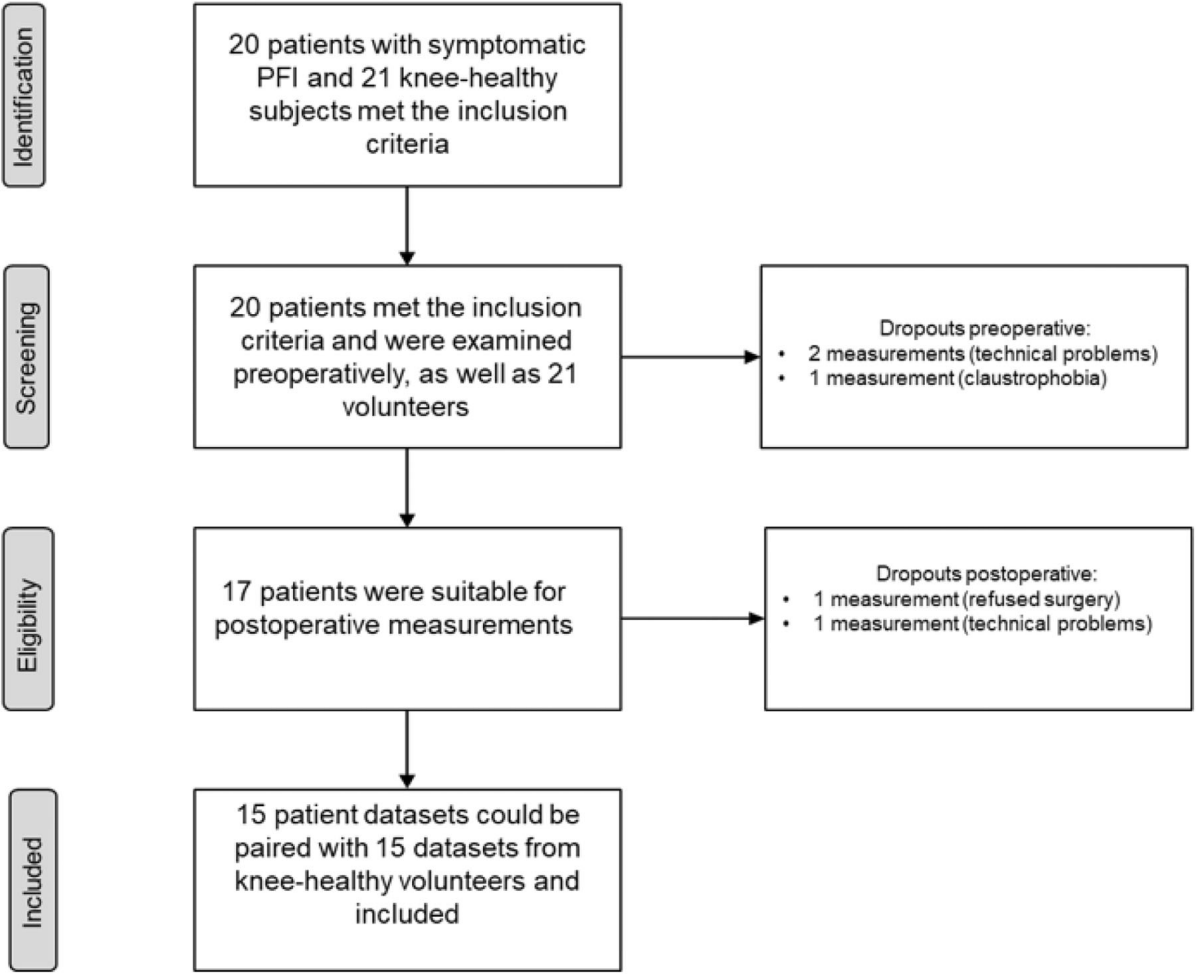
Flow diagram of the selection process.

As a control group, a total of 21 volunteers with clinically inappropriate knee joints without prior surgical interventions were recruited. The same exclusion criteria were applied to the group of volunteers and the patients (see Figure 3).

Both cohorts were matched by the TEA distance, as a metric of knee size with high correlation to the femoral cartilage surface area, and by gender [30].

One study participant showed strong metal artefacts post‐operatively.

### Study participants

#### Patients with PFI

The mean patient age was 27.7 ± 7.5 years (20–49 years). The mean height was 173.3 ± 9.1 cm (160–189 cm) and the mean weight was 71.5 ± 9.1 kg (57–100 kg). The cohort consisted of nine (60.0%) female participants and six (40.0%) male participants.

#### Healthy subjects

The mean age of the subjects with healthy knees was 30.3 ± 6.2 (22–41). The mean height was 174.9 ± 8.2 cm (164–192 cm), and the mean weight was 70.5 ± 8.1 kg (59–86 kg). The cohort consisted of nine (66.7%) female participants and six (33.3%) male participants. Demographic data and anatomic features of both cohorts are listed in Table 1.

**Table 1:** Demographics of the study participants.

	Patients with PFI	*±*SD	Healthy subjects	*±*SD	
*N* =	15		15		
Age (years)	27.7	±7.5	30.3	±6.2	*p* = 0.33
BMI (kg/m^2^)	20.7	±2.5	23.0	±1.8	*p* = 0.52
Height (cm)	173.3	±9.1	174.9	±8.2	*p* = 0.62
Weight (kg)	71.5	±9.1	70.5	±8.1	*p* = 0.75
Side (right/left)	8/7	‐	7/8	‐	
Gender (female/male)^a^	9/6	‐	9/6	‐	
Leg axis (degree)	−0.5	±2.6	‐	‐	
Trochlear dysplasia (Dejour)					
No dysplasia	2	‐	13	‐	
Moderate (Type A/B)	11	‐	2	‐	
Severe Type C/D)	2	‐	0	‐	
TTTG (mm)	11.6	±4.4	5.6	±3.5	
TEA distance (mm)^a^	**77**	**±6**	**78**	±**6**	*p* = 0.26

Abbreviations: BMI, body mass index; PFI, patellofemoral instability; SD, standard deviation; TEA, transepicondylar axis; TTTG, tibial tuberosity to trochlear groove.

^a^ Matching reference.

### Patella shift

In both groups (patients with PFI pre‐ and post‐operative and volunteers), with and without muscle activation through loading (50 N), increasing knee flexion (0–30°) led to increasing medialization of the patella.

The patellar shift is significantly increased in patients with PFI preoperatively compared to volunteers in both loading conditions and across all degrees of flexion.

**Figure 4:**
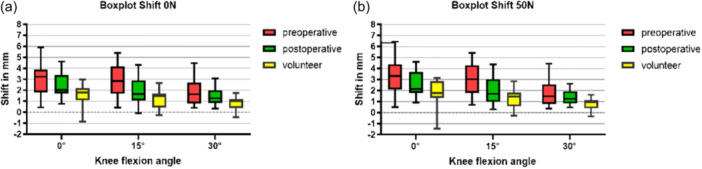
(a) Boxplot of patellar shift in low flexion (0°, 15° and 30°) for PFI patients and volunteers without load. (b) Boxplot of patellar shift during early flexion (0°, 15° and 30°) with axial load (50 N). PFI, patellofemoral instability.

MPFLr reduced the patellar shift, as demonstrated in Figure 4. Statistical significance was reached for 15° flexion with (*p* = 0.007) and without (*p* = 0.011) muscle activation (see Table 2 for unloaded positions and Table 3 for loaded positions). Post‐operatively, the patellar shift is normalized compared to the volunteers as no significant differences are found. Only the comparison in the loaded 30° flexion position shows mild statistical significance (*p* = 0.037).

**Table 2:** Parameters preoperative, post‐operative and volunteers without load.

			Preoperative 0 N, mean ± SD	Post‐operative 0 N, mean ± SD	Volunteers 0 N, mean ± SD	*p*	*p*	*p*
Flexion		*n*=				*p* pre‐ vs. post‐operative	*p* preoperative vs. volunteers	*p* post‐operative vs. volunteers
	Shift (mm)							
0°		15/15	3.04 ± 1.64	2.41 ± 1.11	1.54 ± 1.10	0.124	**0.011**	0.093
15°		15/15	2.75 ± 1.46	1.98 ± 1.21	1.27 ± 0.83	**0.011**	**0.004**	0.123
30°		15/15	1.84 ± 1.21	1.40 ± 0.82	0.76 ± 0.66	0.103	**0.015**	0.077
	Rotation (°)							
0°		15/15	‐	‐	‐	‐	‐	‐
15°		15/15	2.86 ± 2.80	2.19 ± 2.11	1.52 ± 0.85	0.510	0.250	0.813
30°		15/15	6.50 ± 5.85	5.06 ± 2.88	2.65 ± 1.74	0.778	**0.045**	**0.020**
	Tilt (°)							
0°		15/15	24.92 ± 12.08	16.99 ± 11.11	16.20 ± 5.92	**0.008**	0.056	0.652
15°		15/15	23.52 ± 10.61	17.18 ± 10.48	16.31 ± 6.08	**0.006**	**0.041**	0.983
30°		15/15	19.91 ± 9.78	16.49 ± 9.82	15.51 ± 5.51	**0.003**	0.161	0.983

*Note*: Significant *p*‐values < 0.05 are bolded.

**Table 3:** Parameters preoperative, post‐operative and volunteers with axial load (50 N).

			Preoperative 50 N, mean ± SD	Post‐operative 50 N, mean ± SD	Volunteers 50 N, mean ± SD	*p*	*p*	*p*
Flexion		*n*=				*p* pre‐ vs. post‐operative	*p* preoperative vs. volunteers	*p* post‐operative vs. volunteers
	Shift (mm)							
0°		15/15	3.35 ± 1.75	2.59 ± 1.12	1.68 ± 1.26	0.087	**0.010**	0.080
15°		15/15	2.85 ± 1.49	1.93 ± 1.25	1.32 ± 0.83	**0.007**	**0.003**	0.339
30°		15/15	1.69 ± 1.14	1.36 ± 0.68	0.73 ± 0.59	0.221	**0.016**	**0.037**
	Rotation (°)							
0°		15/15	1.94 ± 1.89	0.68 ± 0.96	1.24 ± 2.05	**0.009**	0.081	0.892
15°		15/15	3.07 ± 2.70	2.39 ± 2.28	1.75 ± 1.18	0.382	0.267	0.786
30°		15/15	6.91 ± 6.51	4.86 ± 2.64	3.15 ± 2.05	0.470	0.137	0.077
	Tilt (°)							
0°		15/15	26.62 ± 12.72	16.81 ± 11.58	16.98 ± 6.50	**0.013**	**0.033**	0.363
15°		15/15	24.26 ± 10.52	16.09 ± 10.45	16.21 ± 6.11	**0.005**	**0.024**	0.751
30°		15/15	18.86 ± 9.40	16.29 ± 9.37	14.68 ± 5.58	**0.002**	0.126	0.813

*Note*: Significant *p*‐values < 0.05 are bolded.

Considering the influence of quadriceps muscle activation on the patellar shift, patients with PFI show a preoperative increase in extension (*p* = 0.001) and a statistical trend at 15° flexion (*p* = 0.056). In contrast, a reduction of the shift is observed at 30° flexion (*p* = 0.004). Following patellofemoral stabilization via MPFLr, patients exhibit a pattern similar to that of the volunteers, with a significant difference in extension, whereas no significant differences are observed at 15° and 30° flexion. Table 4 gives an overview of the pre‐ and post‐operative values under loading and unloading.

**Table 4:** Overview of parameters with and without quadriceps activation.

			PrE 50 N vs. PrE 0 N		Post 50 N vs. Post 0 N		Vol 50 N vs. Vol 0 N	
Flexion		*n*=	*Δ*	*p*	*Δ*	*p*	*Δ*	*p*
	Shift (mm)							
0°		15/15	0.31 ± 0.11	**0.001**	0.18 ± 0.01	**0.008**	0.14 ± 0.16	**0.018**
15°		15/15	0.10 ± 0.03	0.056	−0.05 ± 0.04	0.084	0.05 ± 0.00	0.117
30°		15/15	−0.15 ± 0.07	**0.004**	−0.04 ± 0.14	0.346	−0.03 ± 0.07	0.683
	Rotation (°)							
0°		15/15	‐		‐		‐	
15°		15/15	0.21 ± 0.10	0.427	0.20 ± 0.17	0.173	0.23 ± 0.33	0.198
30°		15/15	0.41 ± 0.66	0.173	−0.20 ± 0.24	0.730	0.50 ± 0.31	0.551
	Tilt (°)							
0°		15/15	1.70 ± 0.64	**0.002**	0.18 ± 0.47	0.382	0.78 ± 58	0.061
15°		15/15	0.74 ± 0.09	0.053	−1.09 ± 0.03	0.402	−0.10 ± 0.03	0.776
30°		15/15	−1.05 ± 0.38	**0.001**	−0.20 ± 0.45	0.730	−0.83 ± 0.07	0.084

*Note*: Significant *p*‐values < 0.05 are bolded.

### Patellar rotation

**Figure 5:**
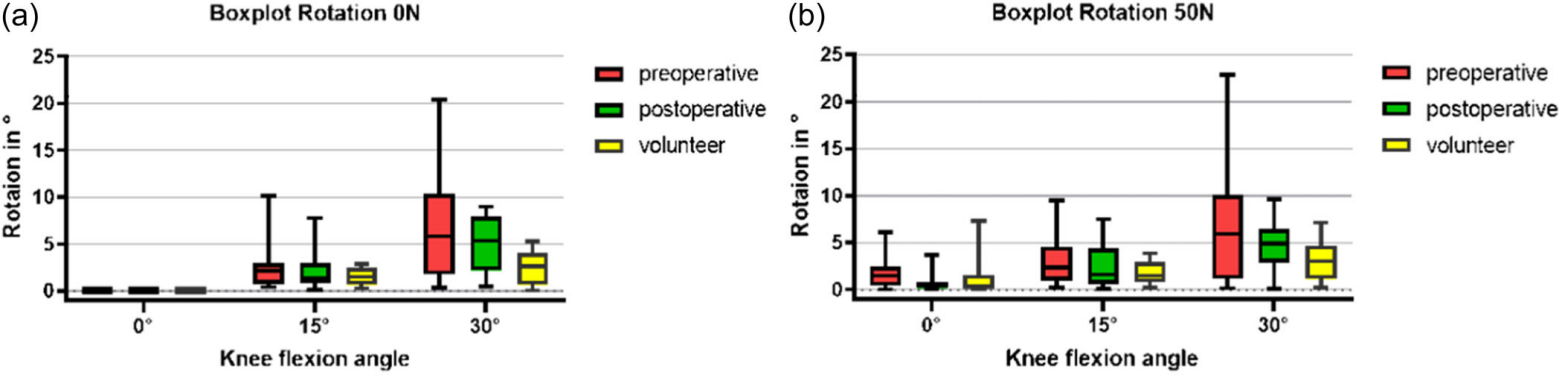
(a) Boxplot of patellar rotation during low knee flexion (0°, 15° and 30°) for PFI patients and volunteers in unloaded situations. (b) Boxplot of patellar rotation during low knee flexion (0°, 15° and 30°) with axial loading (50 N). PFI, patellofemoral instability.

In both groups, with and without loading, increasing lateral patella rotation (positive rotation) was seen accompanied by increasing knee flexion, as can be seen in Figure 5. Patients with PFI show greater patellar rotation compared to the volunteers only in the unloaded 30° flexion position (*p* = 0.045), while no differences are observed in the other measurements. MPFLr reduced patellar rotation only in 0° flexion with axial load, as can be seen in Tables 2 and 3 (*p* = 0.009).

Quadriceps muscle activation failed to induce any changes in the measurements across all loading and flexion positions.

### Patellar tilt

**Figure 6:**
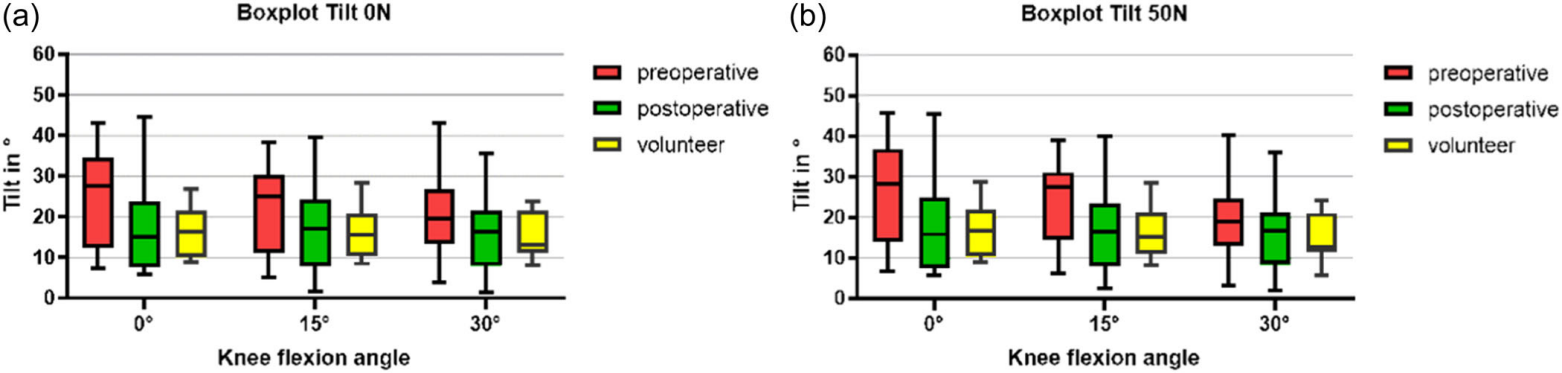
(a) Boxplot of tilt during low flexion (0°, 15° and 30°) for PFI patients and volunteers in loaded and unloaded situations. (b) Boxplot of tilt during low flexion (0°, 15° and 30°) with 50 N axial load. PFI, patellofemoral instability.

In both groups, a decrease in patellar tilt was observed with and without axial load, with increasing knee flexion positions. Patients with PFI exhibited an increased patellar tilt compared to the volunteers at 15° without load (*p* = 0.041), as well as under load in extension (*p* = 0.033) and at 15° flexion (*p* = 0.024). MPFLr reduced patellar tilt in all flexion positions with and without axial load, all *p* values < 0.013 (see Figure 6, Table 3 for unloaded positions and Table 4 for loaded positions). After MPFLr, no differences in volunteers with healthy knees were seen.

Patients with PFI demonstrated an increased influence of quadriceps muscle activation on patellar tilt compared to healthy volunteers. This effect was significantly enhanced under load in extension (*p* = 0.002) and reduced at 30° flexion (*p* = 0.001). Post‐operatively, a similar pattern to that of the volunteers was observed, with no influence of quadriceps activation on patellar tilt.

### Correlation of KPs with CCA

None of the parameters tested showed a strong correlation to the patellofemoral CCA across the different cohorts, flexion and loading situations. Strong correlations with statistical significance were only shown in the patellar rotation post‐operatively in 30° knee flexion with and without muscle activation, as well as in the volunteers in 30° knee flexion with muscle activation (see Table 5).

**Table 5:** Correlations of the kinematic parameters with the patellofemoral cartilage contact area (CCA) over the different flexion and loading situations.

	Shift (in mm)		Rotation (in degree)		Tilt (in degree)		CCA (mean ± SD)	
Flexion	*r*0 N	*r*50 N	*r*0 N	*r*50 N	*r*0 N	*r*50 N	0 N	50 N
Preoperative								
0°	−0.368	−0.339	‐	0.018	−0.068	−0.161	67.33 ± 48.93	72.44 ± 47.46
15°	−0.261	−0.075	−0.293	−0.239	−0.311	0.054	118.86 ± 58.54	112.52 ± 56.81
30°	−0.150	−0.264	−0.275	−0.157	−0.168	−0.246	267.58 ± 99.46	286.81 ± 92.74
Post‐operative								
0°	−0.088	0	‐	0.175	0.203	0.259	155.74 ± 53.85	152.20 ± 55.04
15°	−0.132	−0.032	0.396	0.582*	−0.038	0.009	189.63 ± 64.79	206.61 ± 70.73
30°	−0.086	−0.090	0.574*	0.503*	−0.077	0.182	347.30 ± 54.05	368.71 ± 86.11
Volunteers								
0°	−0.075	−0.132	‐	−0.275	−0.161	−0.139	130.31 ± 68.28	130.71 ± 70.77
15°	−0.371	−0.243	0.100	−0.011	−0.168	−0.232	191.50 ± 92.46	192.57 ± 90.80
30°	0.257	0.584*	0.100	−0.290	−0.079	0.046	370.70 ± 95.09	408.97 ± 76.41

*Note*: The correlation is expressed by means of the correlation coefficient *r*.

Abbreviation: SD, standard deviation.

* Statistically significant (*p* < 0.05).

## DISCUSSION

The main findings of the present study are that isolated MPFLr resulted in a modulation of patellofemoral kinematics both with and without muscle activation, which was particularly evident in patellar shift and tilt. After stabilization using MPFLr, significant differences to healthy knee joint kinematics were only found in patellar rotation in 30° flexion without muscle activation and in patellar shift in 30° flexion with muscle activation. None of the KPs tested showed a strong correlation with the patellofemoral CCA across the different cohorts, flexion and loading situations. Consequently, a single KP did not enable reliable conclusions about patellofemoral CCA. Furthermore, the influence of muscle activation on the different KPs after MPFLr was comparable to that of healthy knees. Based on these findings, passive stabilization through the MPFL seems to be the basic precondition for physiologically active patella stabilization.

Low flexion PFI is characterized by abnormal patellar kinematics in low degrees of flexion [25]. The present study specified these differences and showed a significantly increased patellar shift in patients with PFI for all investigated flexion positions (0°/15°/30°), with and without muscle activation. The patellar tilt of patients with PFI significantly increased in both loading situations at 0° and 15° with load, while there was no statistical difference at 30° flexion. The reason for this observation may be the increasing influence of osseous tracking, especially the trochlear groove which guides the patella predominantly from a knee flexion of about 30°, which can also be seen in patients with low flexion PFI [23]. Differences in patella rotation are only shown in one single tested position: unloaded 30° flexion.

Several recent MRI‐based studies have shown that MPFLr has a positive impact on patellofemoral CCA [25, 26, 28]. The different KPs, however, have not yet been fully elucidated in vivo. The present study suggests that the patellar tilt is a KP that, independent of the loading situation and across all investigated flexion positions, shows a significant change after MPFLr. We observed a significant reduction in all the situations investigated. In comparison to subjects with healthy knees, no difference in tilt was found postoperatively either, with or without muscle activation. MPFLr thus leads to a normalization of the patellar tilt, congruent to current literature [15]. The patellar shift is only significantly medialized by MPFLr at 15° of flexion, with and without muscle activation. In knee extension (0° flexion), the patellar kinematic is not significantly affected by MPFLr in regard to the patellar shift, though the patella is stabilized in its early working range at about 15° flexion and is guided in the low‐angle flexion range in a manner comparable to the patellar kinematics of healthy knees [23]. There were no relevant differences postoperatively in any of the examination situations when compared to healthy knee joints, except in 30° flexion with axial muscle activation. The third investigated parameter, patellar rotation, was only influenced by MPFLr in the loaded extension position. When compared to healthy volunteers, a significant difference was only observed in the unloaded 30° flexion position, which was even slightly increased by MPFLr [19].

It has recently been shown that patients with a low flexion PFI show a reduced influence of muscular stabilization on the patellofemoral CCA when compared to volunteers with healthy knee joints [25]. In the present study, the influence of muscular activation on the different KPs in the patients with PFI was evident in both patellar shift and tilt (each highly significant in extension and 30° flexion, with a statistical trend in 15° flexion). After MPFLr, the influence of muscular activation was only significant for patellar shift in extension. This observation was also seen in comparisons made among the volunteers with healthy knee joints. In PFI patients before MPFLr, muscle activation has a measurable influence on kinematics in contrast to healthy individuals; after surgery, this influence disappears, which can be interpreted as normalization of active patellofemoral stabilization. Static stabilization through MPFLr, or a physiologically functioning MPFL, appears to be the fundamental prerequisite for physiologically active patellar stabilization. This observation could serve as a positive predictive factor for the therapeutic success of isolated MPFLr, particularly in patients who continue to exhibit the described muscle activation pattern following conservative treatment. This warrants further investigation in future studies.

Restoring a physiological patellofemoral congruence can be considered a goal of patellofemoral stabilizing therapies [2, 25, 26, 28]. The measurement of patellofemoral CCA is methodologically complex and not integrated into clinical practice [13, 26]. To investigate whether a less complex kinematic and easy‐to‐measure parameter in clinical practice could be useful for drawing conclusions about the patellofemoral congruence, the correlation of the patellar shift, rotation and tilt to the CCA was evaluated in all the different loading and flexion positions. Even though we found isolated strong correlations of single parameters, no parameter was identified across the different cohorts or across the different knee joint positions and loading conditions which allowed for reliable conclusions to be made about the contact area. Therefore, no conclusions can be drawn from easily measurable parameters regarding patellofemoral congruence and methodologically demanding techniques are still required to calculate the patellofemoral CCA.

### Limitations

A limitation of this study is that it only included a small number of cases (*n* = 15/15). The results should therefore be seen as exploratory and may be different in larger study populations. One of the disadvantages of static‐dynamic MRI, which other studies have also addressed, is the problem of motion artefacts [32]. To reduce this problem, prospective motion correction was used in our setup [13, 16, 32]. It is important to note that the tracking camera used and its limited focal range further restrict the knee flexion angle, which is already limited by the scanner bore. In our experimental setup, however, flexion angles of up to 30° could be realized without any problems, even for large subjects. Since the MPFL seems to have an increasingly smaller influence on patella tracking above 30° of knee flexion, larger flexion angles would probably be of little added value for testing the given hypothesis. In this study, the CNN model was trained only on the automatically segmented bones, not the cartilage. While the automatically segmented bone masks were only indirectly used for a rough alignment of the images, the cartilage masks formed the basis for the calculation of the CCA at each flexion angle, meaning that a much more accurate cartilage segmentation was required. With the existing number of samples in the database, this level of accuracy could not be achieved with an automatic segmentation model. To solve this problem, we used the bone registration described to transfer the manually segmented cartilages from the base image to the images of the knee joint for 15° and 30°.

## CONCLUSION

Isolated MPFLr can normalize patellar shift and tilt in patients with low flexion instability. Considering the influence of muscle activation, passive stabilization through MPFLr seems to be the basic precondition for physiologically active patella stabilization. The investigated KPs as easy‐to‐measure parameters in clinical practice cannot be used to assume normalized CCA for low flexion degrees. Therefore, methodologically demanding methods are still required to calculate the patellofemoral CCA.

## AUTHOR CONTRIBUTIONS

**Kaywan Izadpanah**: Conceptualization; methodology; resources; data curation; investigation; supervision; project administration; funding acquisition. **Markus Siegel**: Conceptualization; methodology; validation; formal analysis; investigation; data curation; writing—review and editing; visualization; software. **Thomas Lange**: Conceptualization; methodology; validation; investigation; resources; data curation; supervision; project administration; funding acquisition; writing—review and editing. **Hans Meine**: Methodology; data curation; software; writing—review and editing. **Elham Taghizadeh**: Methodology; software; formal analysis; visualization; writing—review and editing. **Philipp Maier**: Software; formal analysis; writing—original draft preparation; writing—review and editing; visualization. **Andreas Fuchs**: Writing—review and editing. **Tayfun Yilmaz**: Writing—review and editing. **Hagen Schmal**: Writing—review and editing. All authors have read and agreed to the published version of the manuscript.

## CONFLICT OF INTEREST STATEMENT

The authors declare no conflicts of interest.

## ETHICS STATEMENT

The study was approved by the Institutional Review Board (Ethics Committee of the University of Freiburg, ID 443/16) and entered the German Study Registry (DRKS 00029213). All subjects gave written, informed consent before participating. All participants took part in the study voluntarily and in accordance with the Declaration of Helsinki.

## Data Availability

The authors confirm that the data supporting the findings of this study are available within the article and its Supporting Information.
